# Effects of drought stress on physiological-biochemical characteristics and anatomical structure of *Camellia reticulata* and *C. sasanqua* seedlings: a comprehensive evaluation

**DOI:** 10.3389/fpls.2025.1727605

**Published:** 2025-12-11

**Authors:** Fu-Jun Yan, Yuan-Yuan Huang, Zhi-Yu Zhang, Hong-Xing Xiao, Xue-Qin Wu, Yun-Long Wu, Zhong-Lang Wang, Fang Geng

**Affiliations:** 1 College of Landscape Architecture and Horticulture, Southwest Forestry University, Kunming, China; 2 Yiliang County Zhengyang Camellia Garden Landscaping Co., Ltd, Yiliang, Yunnan, China; 3 Kunming Institute of Botany, Chinese Academy of Sciences, Kunming, China

**Keywords:** *Camellia reticulata*, *Camellia sasanqua*, seedlings, drought stress, comprehensive evaluation

## Abstract

The cultivation and landscaping use of camellias, a valuable woody ornamental, is constrained by its sensitivity to water stress, limiting its adaptability and increasing upkeep expenses. This study investigated the effects of drought stress on leaf anatomical structures and physiological-biochemical indicators in three-year-old seedlings of *Camellia reticulata* and *C. sasanqua*. Seedlings of both camellias were cultivated in 3-gallon nursery pots filled with a 1:1 mixture of red soil and humus soil. The experiment was conducted in a plastic greenhouse maintained at 18 - 28 °C. Drought stress was imposed using the pot water-controlled method, with stress durations of 0, 4, 8, 12, and 16 days. As drought stress intensified, significant phenotypic differences emerged between the two camellias, accompanied by notable changes in leaf tissue structure and physiological-biochemical parameters. Drought differentially affected the stomata of both camellias. Under moderate and severe stress conditions, leaf thickness decreased significantly, with partial stomata closure observed under severe drought. The content of osmoregulatory substances — including soluble sugars, soluble proteins, and proline — increased markedly in both camellias. Concurrently, the activity of antioxidant enzymes exhibited an upward trend, indicating the activation of active defense and adaptation mechanisms under adverse conditions. Finally, comprehensive evaluation of drought tolerance using the subordinate function method and principal component analysis (PCA) yielded scores of D = 0.338 for *C. reticulata* and D = 0.551 for *C. sasanqua*. These results demonstrate that *C. sasanqua* exhibits significantly stronger drought resistance than *C. reticulata*.

## 1 Introduction

With global warming, drought disasters in southern China exhibit expanding spatial distribution, increasing intensity, and atypical characteristics (Huang et al., 2010). Seasonal droughts in this region feature rising frequency due to desynchronization between precipitation and evaporation cycles. The prevalent red soils exhibit poor water retention capacity, accelerating moisture depletion and elevating drought risks (Wen and Chen, 2023). Landscape maintenance often employs extensive management models lacking precise soil moisture monitoring. This irrigation deficit hinders ornamental plants from achieving expected growth and aesthetic performance. Consequently, escalating drought severity coupled with inadequate horticultural practices constitutes the most significant threat to ornamental plant cultivation in southern China. Drought stress triggers complex systemic adaptation mechanisms.

Plants undergo both morphological modifications in roots, stems, and leaves, and cascading physiological-biochemical changes (Ali et al., 2025; Nour et al., 2024; Yu et al., 2023). Leaf thickness serves as a critical drought resistance indicator (Afzal et al., 2017). In response to drought stress, plants modulate structural and physiological traits in their leaves to minimize water loss. Specifically, they can adjust stomatal characteristics—including density, aperture, and opening ratio—to regulate transpiration rates, thereby effectively adapting to arid conditions (Agurla et al., 2018). Under severe drought, when stomatal transpiration declines, cuticular transpiration becomes the dominant water loss pathway. Cuticular wax thickness critically regulates this process (Chen et al., 2011). Plant epicuticular waxes — complex mixtures of very-long-chain fatty acids and derivatives—play vital roles in abiotic stress response (Goodwin and Jenks, 2005; Xue et al., 2017). *Arabidopsis thaliana* demonstrates enhanced wax deposition under drought, representing a water conservation strategy (Patwari et al., 2019).

Despite adaptive adjustments, drought impairs photosynthesis through: stomatal closure reducing CO_2_ availability, compromised chloroplast function, and declining chlorophyll content (Ghotbi-Ravandi et al., 2014). Research has demonstrated that *C. sinensis* exhibits high sensitivity to drought stress, which significantly impacts its photosynthetic physiology, stomatal behavior, and leaf antioxidant system, consequently inhibiting normal plant growth. As drought intensity increases, a substantial accumulation of reactive oxygen species (ROS) occurs within the plant, potentially triggering lipid peroxidation and oxidative damage to cellular components. To mitigate this oxidative pressure, the tea plant activates its antioxidant defense system, significantly upregulating the activities of key antioxidant enzymes such as superoxide dismutase (SOD), peroxidase (POD), and catalase (CAT). This enhanced enzymatic activity effectively scavenges excess free radicals, thereby maintaining cellular redox homeostasis and physiological metabolic stability (Qian et al., 2018). Furthermore, under drought conditions, plants accumulate osmoprotectants—including soluble sugars, proline, fatty acids, and dehydrins—to lower cellular osmotic potential. This mechanism helps maintain cell turgor pressure, safeguards membrane structure and protein integrity, and concurrently supplies energy and carbon skeletons for metabolic processes, thereby enhancing drought adaptation. These osmoprotectants also contribute to sustaining cell turgor and stomatal conductance, which facilitates CO_2_ uptake in leaves and water absorption by roots (Poormohammad Kiani et al., 2007). Thus, the physiological responses and adaptation mechanisms of plants to drought stress are complex and systemic.

Camellias refers collectively to all ornamental plants within the genus *Camellia* of the family Theaceae. Major species include *C. reticulata*, *C. japonica*, *C. sasanqua*, *C. chrysantha*, and *C. azalea*. As one of China’s top ten traditional flowers, camellias are not only high valued for their ornamental appeal but also deeply rooted in cultural heritage, enjoying widespread popularity worldwide (Chen et al., 2020; Xiao et al., 2025; Zhang et al., 2022). Despite their diverse varieties and high aesthetic value, camellias require meticulous management in landscape applications. Drought represents a critical environmental constraint that may impose greater limitations on plant growth than any other stressor (Farooq et al., 2009). Thus, gaining an in-depth understanding of the drought resistance mechanisms in camllias is particularly crucial. In landscape applications, *C. japonica* and *C. sasanqua* are predominantly utilized due to their: cultivation advantages (amenable to pruning and shaping), enhanced resilience (strong disease/pest resistance), low maintenance (minimal management demands). Conversely, *C. reticulata* faces significant constrains: stringent cultivation requirements (demanding specific growth conditions), prohibitive costs (excessively high maintenance expenses for large-scale plantings), and limited application, which severely restrict its landscape utilization. Therefore, this study employs a comprehensive analysis of the differential responses to drought stress between *C. sasanqua* and *C. reticulata*, integrating comparative assessment of leaf anatomical adaptations with multidimensional evaluation of physiological indicators. The objectives are to delineate their distinctive drought-tolerant characteristics and elucidate the underlying physiological mechanisms in response to water deficit, thereby establishing a theoretical foundation for further exploration and enhancement of drought resistance in *Camellia* species.

## 2 Materials and methods

### 2.1 Study area and plant materials

The experimental area is located in the Arboretum of Southwest Forestry University, within Panlong District, Kunming City, Yunnan Province, at an elevation of 1,892 m and coordinates 25°06′ N, 102°76′ E. Kunming experiences a subtropical plateau monsoon climate characterized by distinct dry and wet seasons. The wet season features concentrated rainfall, often in the form of heavy rain and rainstorms. The experiment was conducted in March 2024, during which the average temperature was 22°C, with extreme highs of 27°C and extreme lows of 6°C. The entire month was characterized by sunny and rainless conditions, with a mild climate, long sunshine duration, high ultraviolet intensity, and a large diurnal temperature range.

Three year-old seedlings of *C. reticulata* and *C. sasanqua* with consistent growth status, similar morphological characteristics, and healthy, disease-free conditions were selected for the experiment. Potted seedlings were cultivated in a self-prepared mixed substrate composed of typical southern red soil and humus soil at a 1:1 volume ratio.

### 2.2 Experimental design

The experimental materials were transferred to a plastic greenhouse with uniform light and temperature conditions to exclude natural precipitation and impose drought stress. For each *Camellia* species, 15 pots of healthy and uniformly growing seedlings were selected and subjected to natural drought treatment by withholding irrigation. The drought stress period lasted for 16 days. Soil water content was measured at 16:30 on day 0 (control), day 4 (T1), day 8 (T2), day 12 (T3), and day 16 (T4) of each treatment. Volumetric soil water content was monitored using an HH2 portable soil moisture meter (Delta-T Devices, Cambridge, UK). Measurements were taken every 4 days, with 6–8 randomly selected pots per plant species, and three replicates per pot to ensure data representativeness and accuracy.

### 2.3 Sample collection and parameter quantification

#### 2.3.1 Sample collection

From each replicate, three mature functional leaves were collected. Three of these leaves were fixed in FAA and electron microscopy-specific fixative, respectively, and stored at 4°C in the dark for subsequent anatomical and ultrastructural observations. Another three fresh leaves from the same batch were immediately wrapped in aluminum foil, flash-frozen in liquid nitrogen, and then transferred to an -80°C ultra-low temperature freezer for long-term storage until physiological assays.

#### 2.3.2 Determination of volumetric soil water content and leaf relative water content

The volumetric soil water content for both camellias was measured using a portable soil moisture meter. Measurements were taken once every 4 days, prior to sampling. Simultaneously, the leaf relative water content (RWC) was determined according to standard protocols. Mature leaves from the top of each plant were selected, and three fully expanded leaves were excised. Fresh leaf samples were first weighed to determine the fresh weight (FW), followed by immersion in distilled water until full turgidity (approximately 12 h) to obtain the turgid weight (TW). The saturated leaves were then oven-dried at 75°C for approximately 36 h until constant weight was achieved, yielding the dry weight (DW). The leaf relative water content (RWC) was calculated according to He et al. (2020) using the following formula: 


$$RWC=\frac{\left(FW-DW\right)}{\left(TW-DW\right)}\times 100\%$$

Where: RWC is the leaf relative water content (%); FW is the initial mass of the leaf sample; DW  is the mass after oven-drying; TW is the mass after full hydration.

#### 2.3.3 Preparation and observation of leaf cell microscopy samples

Leaf samples were collected from the third to fifth leaves from the apex of both camellias at each experimental time points. Sections of approximately 1 mm × 1 mm were excised from areas adjacent to the midvein in the central portion of the leaves. These samples were immediately placed in FAA fixative and stored at 4°C. The materials were fixed in FAA for at least 24 hours. After proper preparation, the samples were dehydrated through an ethanol and xylene series, followed by wax infiltration, embedding, and sectioning (section thickness: 4 – 8 μm). The sections were then stained with safranin and fast green. Subsequently, the stained sections were cleared in pure xylene for 5 minutes and mounted with neutral balsam. Observation and photography were performed using a Japanese Olympus CX-21 optical microscope. Based on observations of leaf cross-sections, the following parameters were measured according to the method as follows (He et al., 2020): leaf thickness (mm), upper epidermis thickness (mm), lower epidermis thickness (mm), and palisade tissue thickness (mm). Leaf compactness, sponginess, and the ratio of palisade to spongy tissue were calculated with the following formulas:


$$CTR=\frac{PT}{Lth}\times 100\%$$

$$SR=\frac{ST}{Lth}\times 100\%$$

Where: CTR is the leaf compactness (%); PT is the palisade tissue thickness; Lth is the leaf thickness; SR is the leaf looseness (%); ST is the spongy tissue thickness.

#### 2.3.4 Preparation and observation of leaf samples for electron microscopy

Samples were fixed in 2.5% glutaraldehyde for 2–4 hours, rinsed three times with phosphate buffer, and then post-fixed with 1% osmium tetroxide at 4°C for 2 hours. After three washes with ddH_2_O, the samples were dehydrated through an ethanol gradient series, transitioned with propylene oxide, and infiltrated with 812 resin through a gradient series before embedding. Polymerization was carried out at 60°C. The embedded blocks were sectioned using a Leica UC7 ultramicrotome for semi-thin positioning and ultra-thin sectioning. The sections were double-stained with uranyl acetate and lead citrate, and the ultrastructure organization was observed under a transmission electron microscope. Based on scanning electron microscopy (SEM) images, the following parameters were determined according to the method as follows (He et al., 2020): stomatal density per mm², stomatal length and width, pore length and width, stomatal aperture, and the proportion of open stomata. These parameters were subsequently calculated using the following formulas, respectively:


$$SA=SL\times SW\times \frac{\pi }{4}$$

$$SAR=\frac{SD}{SC}$$

Where: SA is the stomatal aperture; SL is the stomatal opening length; SW is the stomatal opening width; SAR is the stomatal opening rate (%); SD is the number of open stomata per unit area; SC is the total number of stomata.

#### 2.3.5 Measurement of physiological indices

Soluble sugar content in the leaves was determined by the obarbituric acid method (Palma et al., 2009). The 1 mL of extraction solution and 5 mL of 0.5% thiobarbituric acid were added, boiled in a water bath for 10 mins, quickly cooled, and centrifuged at 12,000 rpm for 10 mins. The supernatant was taken and using 0.5% thiobarbituric acid as blank. The absorbance values were recorded using a UV–Vis spectro photometer at 450 nm.

Soluble protein content was measured via the Coomassie Brilliant Blue G-250 method (Wang and Huang, 2006). Using Coomassie Bril liant Blue G250 dye. Adding 1 mL of the sample extraction solution in a test tube, then 5 mL of Coomassie Brilliant Blue reagent was added, mixed thoroughly, and left undisturbed for 2 mins. Then the absorbance of the supernatant was measured at 595 nm. The standard curve was checked according to the absorbance value to determine the protein content in the sample.

Free proline content was quantified using the acid ninhydrin method (Zhang et al., 2015). In this procedure, 2 mL of acid ninhydrin reagent and 2 mL of glacial acetic acid were added to 1 mL of extraction solution. The mixture was incubated at 100°C for 60 mins, then cooled in an ice bath before adding 4 mL of toluene. The absorbance of the toluene layer was measured at 520 nm using a spectrophotometer.

The relative chlorophyll content of leaves was measured using a SPAD-502 Plus chlorophyll meter (Konica Minolta, Japan). The instrument was calibrated before measurement. Avoiding the midrib, three points were selected at approximately 2/3 of the distance from the petiole in the middle part of the leaf for measurement, and the average value was taken as the SPAD value for that leaf, thereby reducing errors caused by uneven chlorophyll distribution.

The malondialdehyde (MDA) content, as well as the enzymatic activities of superoxide dismutase (SOD), peroxidase (POD), and catalase (CAT), were assayed using specific detection kits (Suzhou Comin Biotechnology Co., Ltd., China).

### 2.5 Statistical analysis

Data were analyzed using Microsoft Excel 2019, IMAGE J software, and IBM SPSS Statistics 26. One-way ANOVA was employed to examine significant differences among treatment groups. Pearson correlation analysis was conducted for 16 indicators using Origin software. The drought resistance of the two camellias was evaluated by calculating subordinate function values (R) according to the formula (Ji et al., 2024), where W(j) is the weight of the j-th indicator and D represents the comprehensive evaluation index.


$$R\left(X_{i}\right)=\left(X_{i}-X_{\text{min}}\right)\times \left(X_{\text{max}}-X_{\text{min}}\right)$$

$$R\left(X_{i}\right)=1-\left(X_{i}-X_{\text{min}}\right)\times \left(X_{\text{max}}-X_{\text{min}}\right)$$

Where: $X_{i}\;$ represents the measured value of an indicator; $X_{\min}\;$ is the minimum value of that indicator; $X_{\max\;}$ is the maximum value of that indicator; If an indicator is negatively correlated with drought resistance, the anti-subordinate function should be applied for conservation.


$$\text{W}\left(\text{j}\right)=V_{j}/\sum_{j=1}^{n}\text{Vj},\text{ j}=1,2,3,\ldots ,\text{n}$$

Where: W(j)  is the weight of the j-th indicator, and $V_{j}$ is the contribution rate of the j-th indicator for each material.


$$\text{D}=\sum_{j=1}^{n}\left[\text{X}\left(\mu \right)\times \text{Wj}\right],\text{ j}=1,2,3,\ldots ,n$$

Where: The D-value represents the comprehensive evaluation index of drought resistance under drought stress based on multiple indicators.

## 3 Results and analysis

### 3.1 Morphological and hydrological responses of two *Camellia* species to drought stress

**Figure 1:**
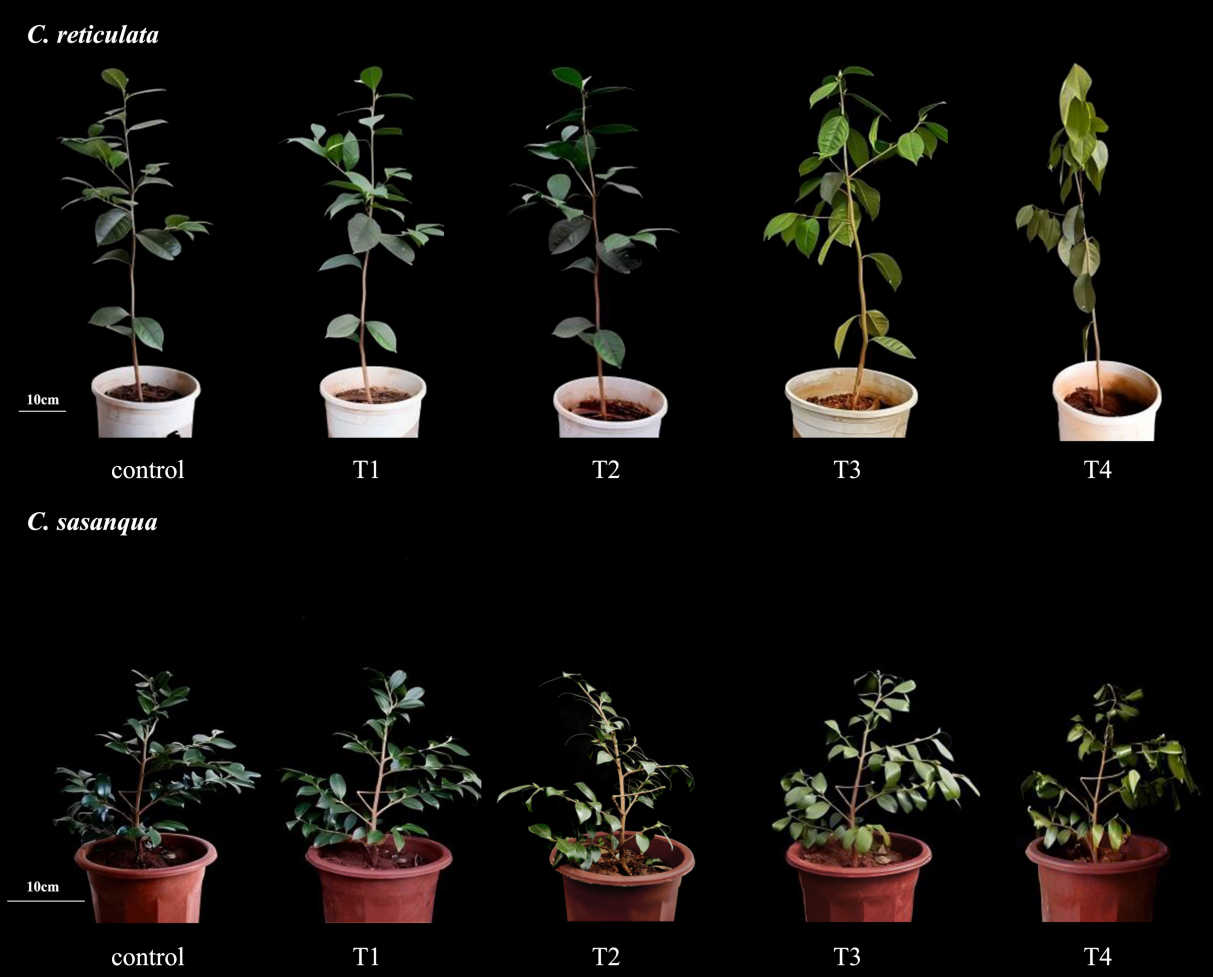
Morphological changes in two camellias under drought stress. Control, T1, T2, T3, and T4 represent the 0 day, 4^th^ day, 8^th^ day, 12^th^ day, and 16^th^ day respectively.

Under drought stress, the morphological characteristics of both camellias underwent significant alterations (**Figure 1**). Under well-watered conditions, both *Camellia* species exhibited dark green glossy leaves and vigorous new shoots. No phenotypic changes were observed after 4 days of drought treatment. By day 8, leaf glossiness decreased in both species, with *C. reticulata* showing wilting symptoms and *C. sasanqua* developing leaf yellowing. At day 12, *C. reticulata* demonstrated significant wilting with chlorosis, while *C. sasanqua* displayed leaf curling and pronounced yellowing. By day 16, *C. reticulata* plants showed severe wilting, complete loss of luster, and approached mortality, whereas *C. sasanqua* maintained viability despite severe symptoms including deeply curled leaves, necrotic yellowing, brittle texture, and stem inclination.

**Figure 2:**
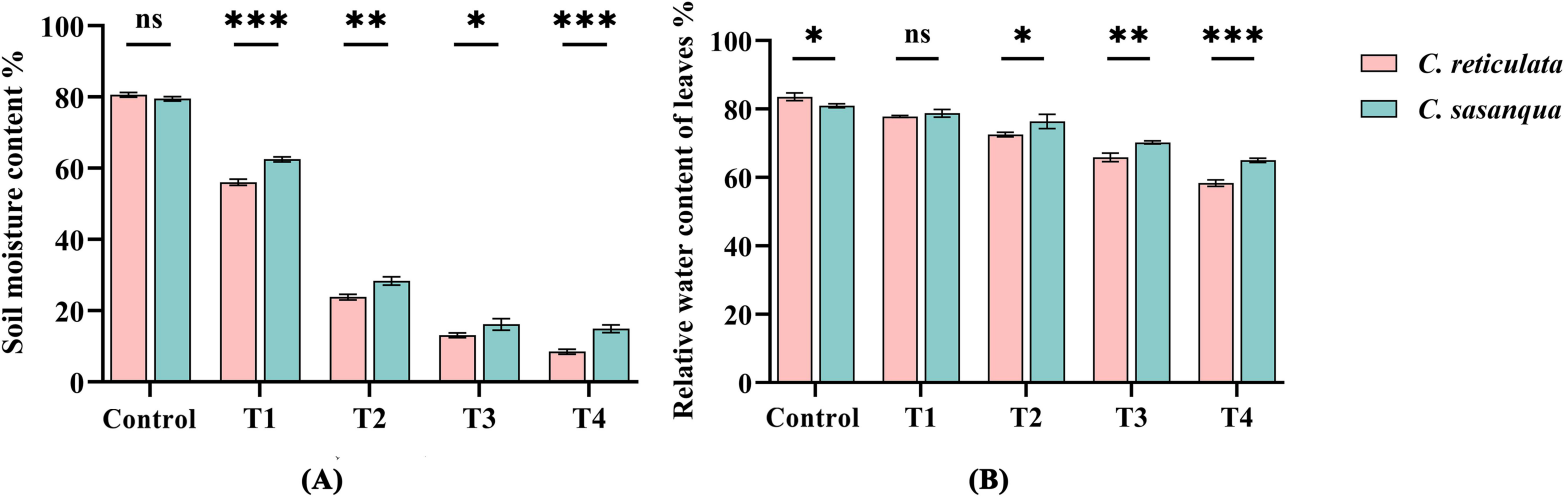
Water content under different drought conditions: **(A)** soil water content; **(B)** relative water content of leaves. Value indicated the means ± SD (n = 3). *P < 0.05, **P < 0.01, ***P < 0.001 (Student’s t test). ns: “not significant”.

Under drought stress, soil water content progressively decreased in both *Camellia* species (**Figure 2A**). At T4 stage, *C. reticulata* exhibited significantly lower soil moisture (8.4%) than *C. sasanqua* (16.3%). Notably, *C. reticulata* maintained significantly reduced soil water content compared to *C. sasanqua* at all stress stages except the well-watered control. Drought stress significantly reduced leaf relative water content (RWC) in both *Camellia* species (**Figure 2B**). Although *C. reticulata* initially showed a higher RWC (83.7%) than *C. sasanqua* (81.0%) at the control stage, its RWC became consistently and significantly lower from T2 onward. At the T4 stage, RWC values declined to 58.4% in *C. reticulata* and 65.0% in *C. sasanqua*.

### 3.2 Leaf anatomical structure

#### 3.2.1 Changes in leaf anatomical structure of two camellias under drought stress

**Figure 3:**
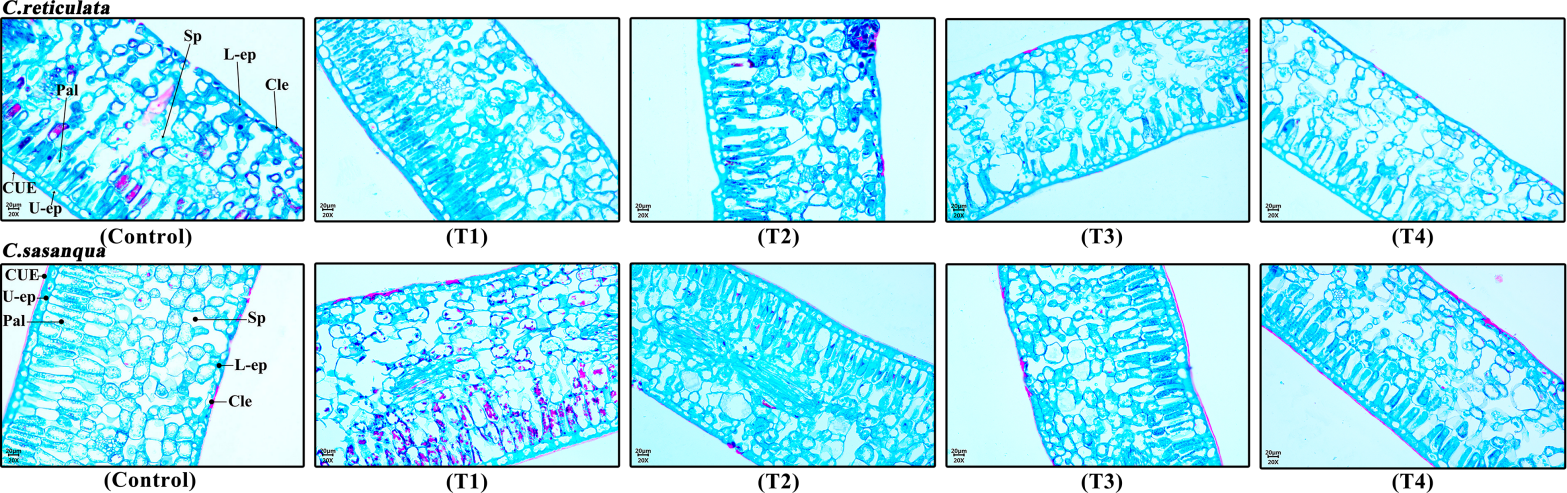
Morphological changes in leaf tissue architecture of *C. reticulata* and *C. sasanqua* in response to drought stress. Cross-sectional anatomical structure of *Camellia* leaves. T1, T2, T3, T4, and control represent the 4th, 8th, 12th, 16th days, and natural condition (control), respectively. U-ep, Upper epidermis; L-ep, Lower epidermis; CUE, Cuticle of upper epidermis; CLE, Cuticle of lower epidermis; Pal, Palisade tissue; Sp, Spongy tissue.

Leaf anatomical structures of the two *Camellia* species under drought stress are shown in **Figure 3**. The leaves of both camellias exhibited typical dorsiventral structure: the palisade tissue adjacent to the upper epidermis consisted of one to two layers of tightly arranged oblong to elliptical cells; while the spongy tissue beneath the palisade layer comprised nearly spherical cells with relatively loose arrangement and large intercellular spaces. As drought stress intensified, significant changes occured in the leaf anatomical structure of both camellias. By the final stress stage (T4), the spongy tissue of both camellias showed varying degrees of loosened arrangement, characterized by more dispersed cells and enlarged intercellular spaces. However, *C. sasanqua* maintained a relatively compact spongy tissue structure with densely arranged cells, whereas *C. reticulata* exhibited more pronounced loosening with noticeably disrupted cellular organization.

**Figure 4:**
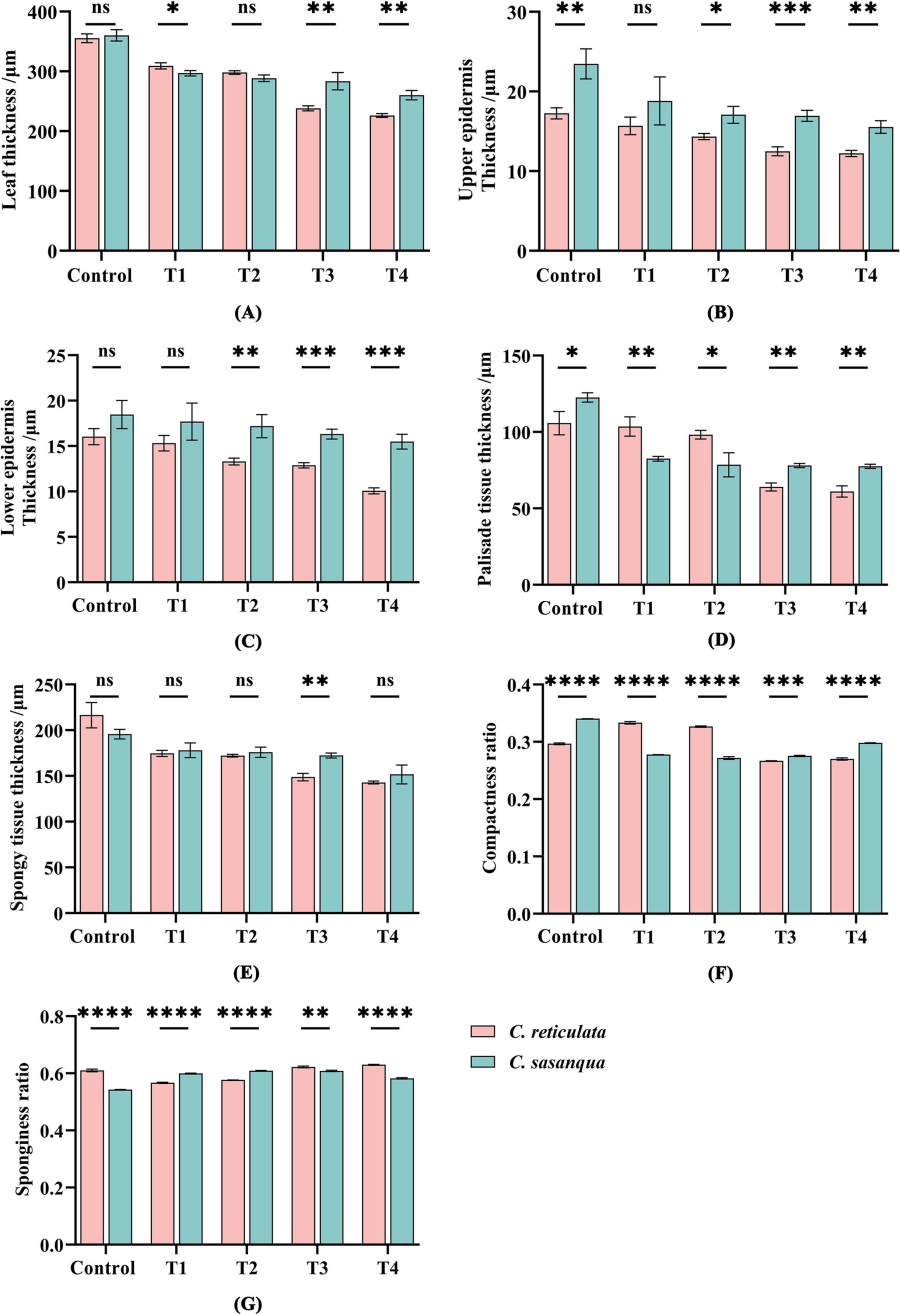
Characteristic parameters of leaf anatomical structure under drought stress. **(A)** Leaf thickness; **(B)** Leaf upper epidermis thickness; **(C)** Leaf lower epidermis thickness; **(D)** Palisade tissue thickness; **(E)** Compactness ratio; **(F)** Sponginess ratio; **(G)** the leaf looseness. Value indicated the means ± SD (n = 3). *P < 0.05, **P < 0.01, ***P < 0.001, ****P < 0.0001 (Student’s t test). “not significant”.

Under progressive drought stress, both *Camellia* species showed consistent reductions in leaf anatomical parameters with significant interspecific variations (**Figure 4**). Leaf thickness exhibited significant interspecific differences at all stages except control and T2 (**Figure 4A**). Upper epidermal thickness varied significantly except at T1, while lower epidermal thickness showed non-significant differences only at control and T1 stages (**Figures 4B, C**). Palisade tissue thickness demonstrated significant interspecific differences throughout all stress periods, whereas spongy tissue thickness varied significantly only at T3 (**Figures 4D, E**). Both compactness and sponginess ratios showed significant interspecific differences across all stages (**Figures 4F, G**).

#### 3.2.2 Changes in leaf stomatal morphology of two camellias under drought stress

**Figure 5:**
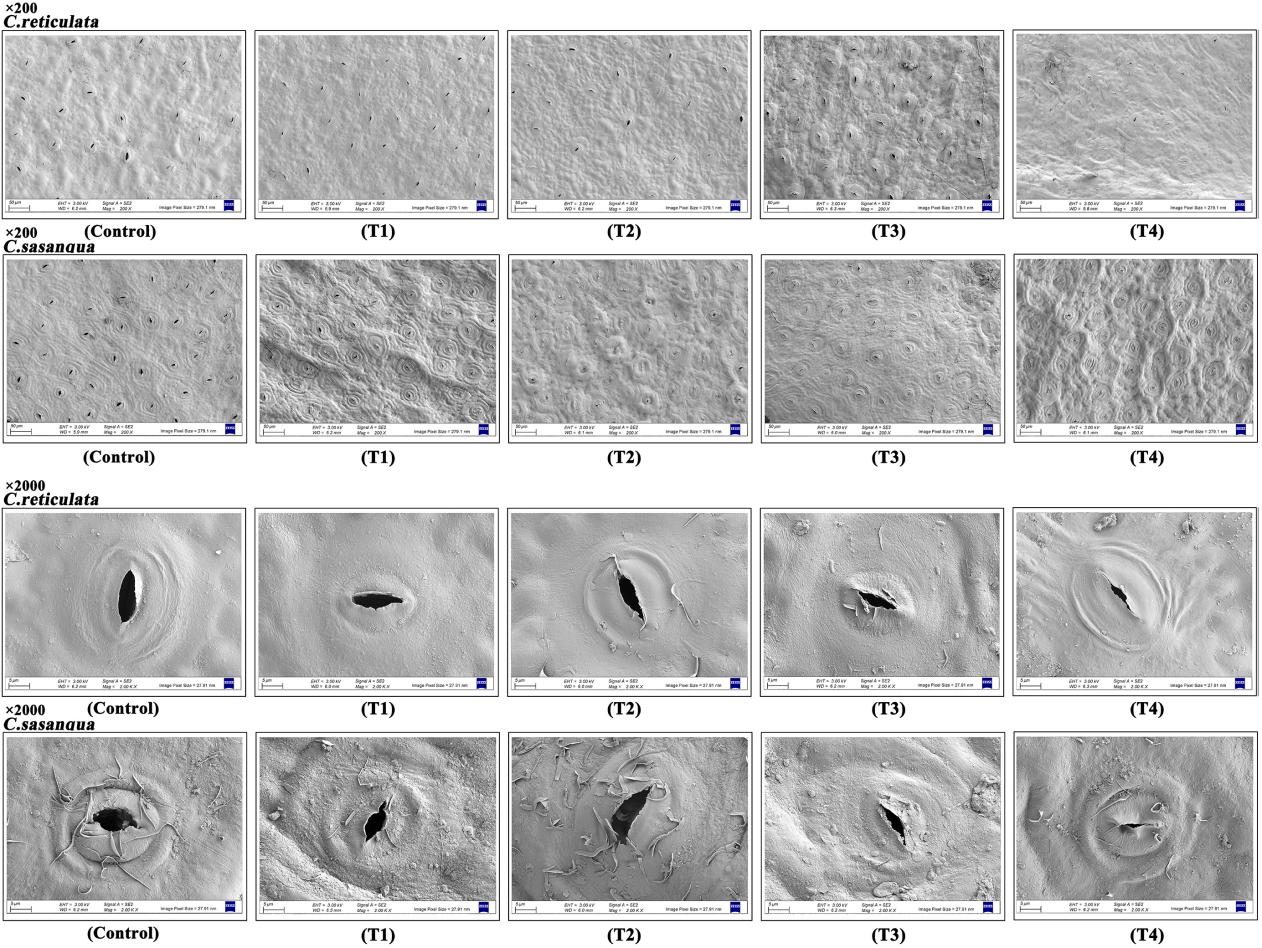
Effects of drought stress on leaf stomatal morphology in two camellias (200X and 2000X). T1, T2, T3, T4, and control represent data at Day 4, Day 8, Day 12, Day 16, and natural condition (control), respectively.

Both *Camellia* species exhibited stomata exclusively on the lower epidermis (**Figure 5**). Drought stress induced stomatal closure and epicuticular wax deposition, with species-specific progression patterns. *C. reticulata* developed stomatal depression and wax accumulation from T2, showed maximal structural deformation at T3, and reached near-complete stomatal closure by T4. In contrast, *C. sasanqua* initiated wax deposition as early as T1, achieved peak accumulation with substantial stomatal closure at T2, and maintained closed stomata through T3-T4 despite subsequent wax reduction.

**Figure 6:**
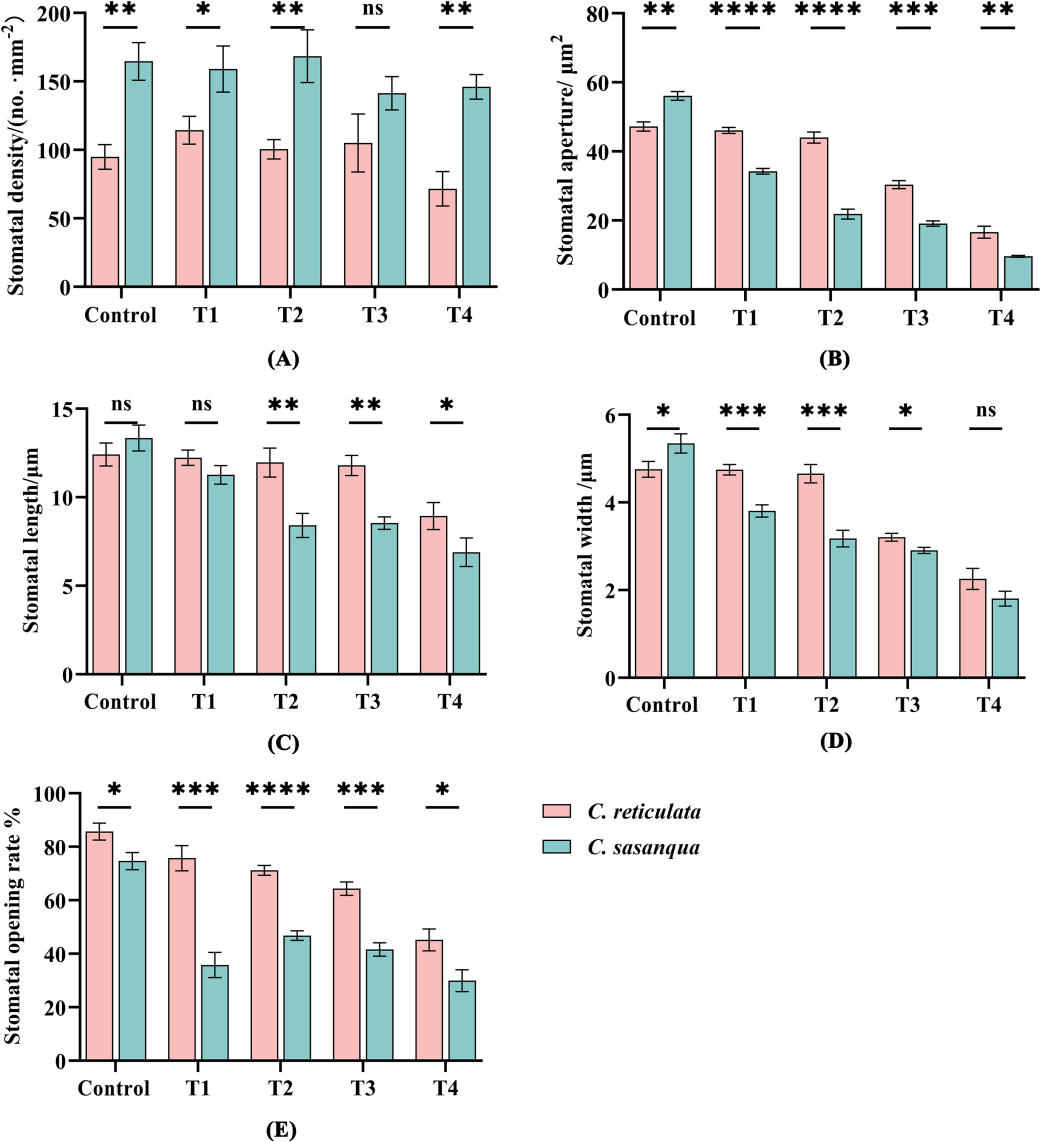
Leaf stomatal characteristic parameters under drought stress. **(A)** Stomatal density; **(B)** Stomatal aperture; **(C)** Stomatal length; **(D)** Stomatal width; **(E)** Stomatal opening rate. Value indicated the means ± SD (n = 3). *P < 0.05, **P < 0.01, ***P < 0.001, ****P < 0.0001 (Student’s t test).

Drought stress significantly modified stomatal features in both *Camellia* species (**Figure 6**). Stomatal density showed contrasting patterns, with *C. reticulata* exhibiting an initial increase followed by decrease, while *C. sasanqua* demonstrated a continuous decline; significant interspecific differences were observed at all stages except T3 (**Figure 6A**). Stomatal aperture size progressively decreased in both species with significant differences throughout the stress period (**Figure 5B**). Both stomatal length and width generally decreased under intensified drought, with non-significant interspecific differences in length only at control and T1 stages, while width showed significant differences at all stages except T4 (**Figures 6C, D**). Stomatal opening ratio displayed distinct response patterns: *C. reticulata* showed continuous decline with prolonged stress, whereas *C. sasanqua* exhibited an immediate significant reduction from control to T1, temporary recovery at T2, followed by progressive decrease (**Figure 6E**).

#### 3.2.3 Effects of drought stress on the microstructure of leaf surface wax in two *Camellia* species

**Figure 7:**
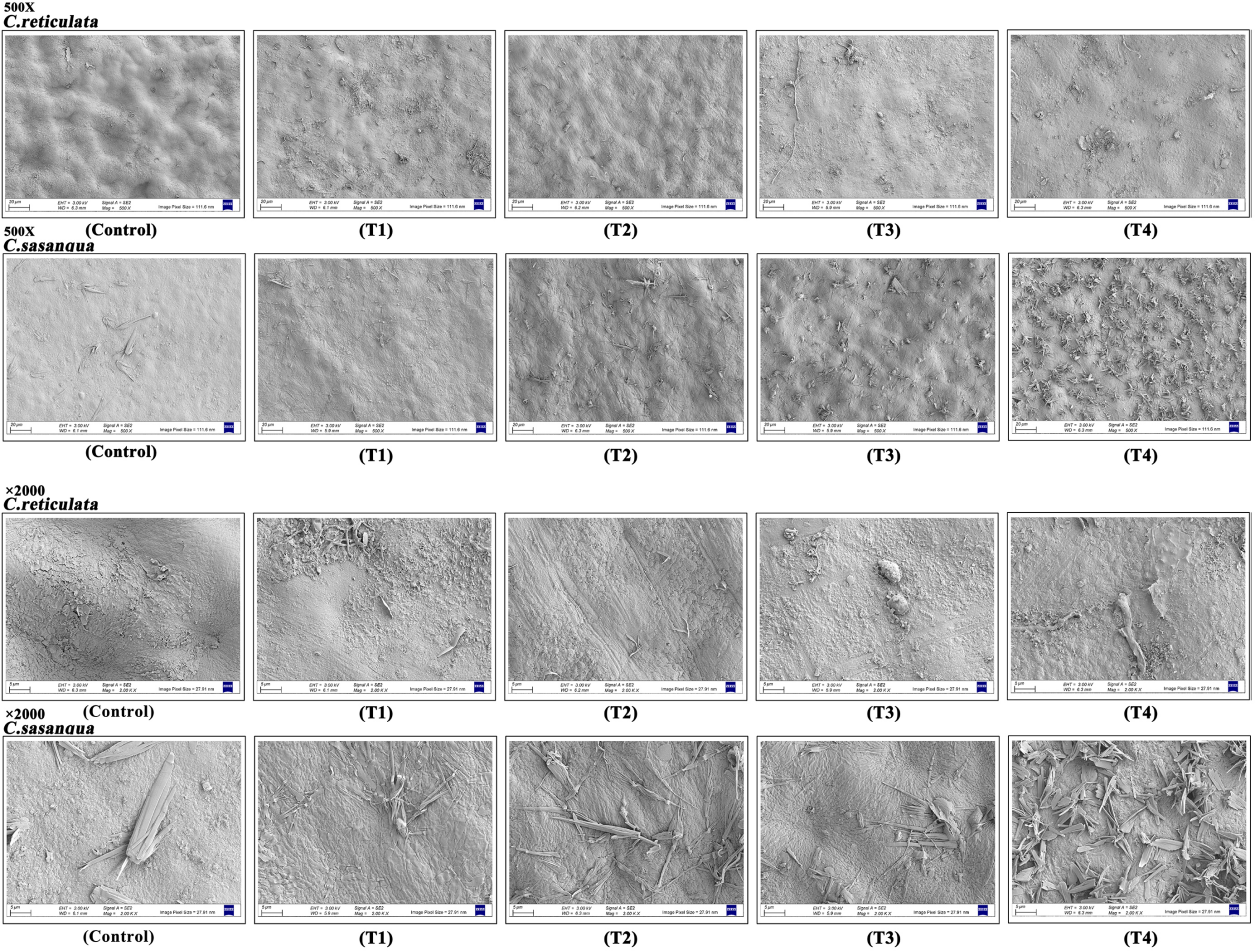
Effects of drought stress on the morphology of leaf surface wax in two camellias (500X and 2000X). T1, T2, T3, T4, and control represent data at Day 4, Day 8, Day 12, Day 16, and natural condition (control), respectively.

Both *Camellia* species developed epicuticular wax coverage, with consistently higher deposition rates in *C. sasanqua* than *C. reticulata* (**Figure 7**). *C. reticulata* exhibited abundant clustered and honeycomb-like wax crystals at T1, followed by reduced coverage during T2-T3 and subsequent re-accumulation at T4. In contrast, *C. sasanqua* displayed minimal striated wax distribution at T1 but demonstrated progressive accumulation from T2 onward, reaching peak deposition with dense striated structures at T4.

### 3.3 Leaf physiology and biochemistry

**Figure 8:**
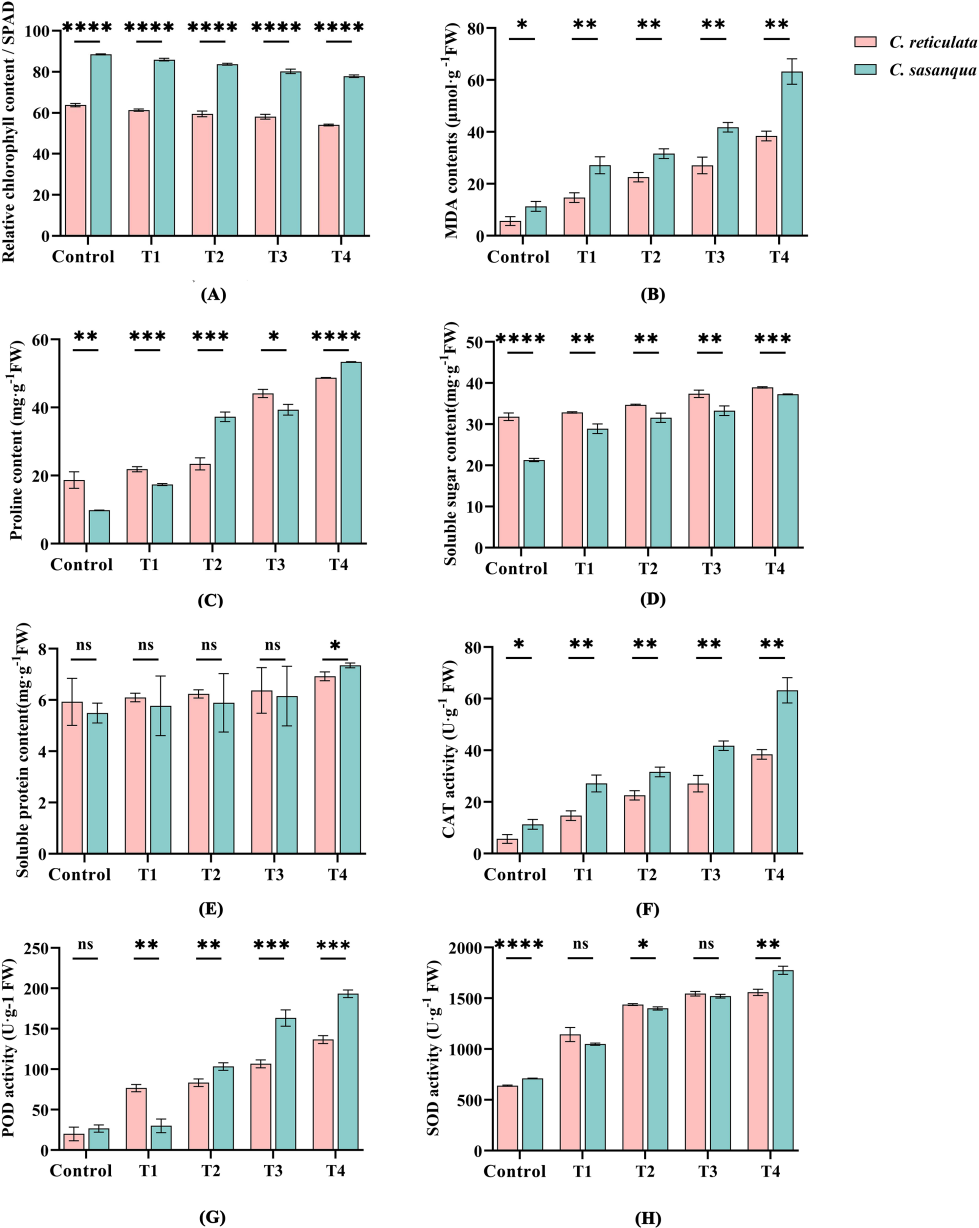
Two camellias under different drought conditions. **(A)** Relative Chlorophyll content; **(B)** MDA content; **(C)** Relative proline content; **(D)** Soluble sugar content; **(E)** Soluble protein content; **(F)** Catalase (CAT) activity; **(G)** Peroxidase (POD) activity; **(H)** Superoxide dismutase (SOD) activity. Value indicated the means ± SD (n = 3). *P < 0.05, **P < 0.01, ***P < 0.001, ****P < 0.0001 (Student’s t test).

As shown in **Figure 8**, progressive drought stress significantly decreased relative chlorophyll content in both species, with consistently higher values in *C. sasanqua* across all stages (**Figure 8A**). Malondialdehyde (MDA) content increased with stress intensity, showing significantly greater accumulation in *C. sasanqua* throughout the experiment (**Figure 8B**). While proline, soluble sugars, soluble proteins, and antioxidant enzymes (CAT, POD, SOD) all exhibited increasing trends, species-specific variations emerged: soluble sugar content remained significantly higher in *C. reticulata* (**Figure 8D**), whereas CAT activity was consistently superior in *C. sasanqua* (**Figure 8F**). Proline levels were initially higher in *C. reticulata* during control and T1 stages but became significantly elevated in *C. sasanqua* from T2 onward (**Figure 8C**). Soluble protein content showed no significant interspecific differences until T4, when *C. sasanqua* demonstrated markedly higher levels (**Figure 8E**). POD activity was lower in *C. reticulata* during control, peaked at T1, but was subsequently exceeded by *C. sasanqua* from T2 (**Figure 8G**). SOD activity was initially higher in *C. reticulata* but became significantly greater in *C. sasanqua* at T4 (**Figure 8H**).

### 3.4 Comprehensive evaluation of drought resistance in two *Camellia* species under drought stress

**Figure 9:**
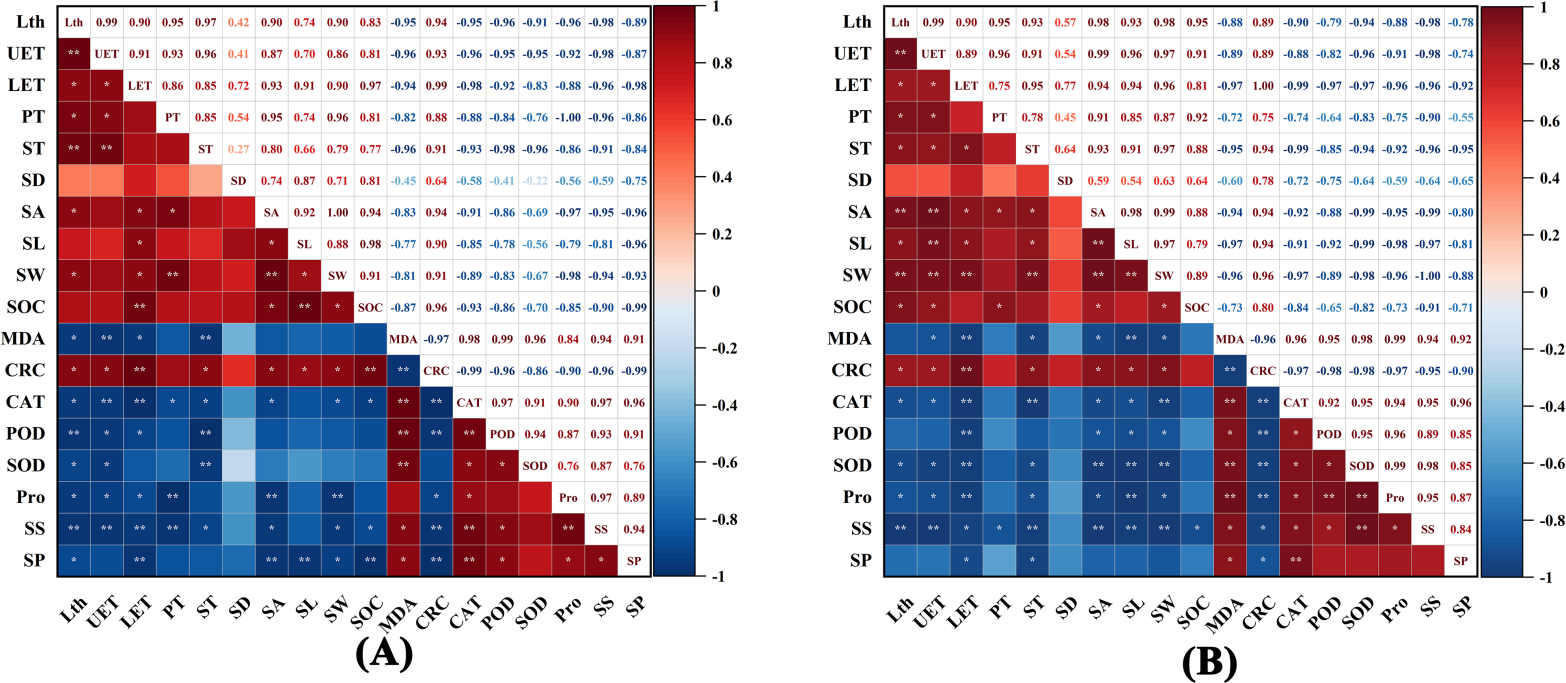
Correlation coefficients of parameters in two *Camellia* species under drought stress. **(A)***C*. *reticulata*; **(B)***C*. *sasanqua*. Lth, Leaf thickness; UET, Upper epidermis thickness; LET, Lower epidermis thickness; PT, Palisade tissue thickness; ST, Spongy tissue thickness; SD, Stomatal density; SA, Stomatal aperture; SL, Stomatal length; SW, Stomatal width; SOC, Stomatal opening count; MDA, Malondialdehyde; CRC, Chlorophyll relative content; CAT, Catalase; POD, Peroxidase; SOD, Superoxide dismutase; Pro, Proline; SS, Soluble sugar; SP, Soluble protein. Red indicates positive correlation, blue indicates negative correlations. darker colors represent more significant correlations. *P < 0.05, **P < 0.01.

Correlation analysis of all parameters in both *Camellia* species revealed systematic associations between leaf structural and physiological-biochemical indicators (**Figure 9**). In *C. reticulata*, leaf thickness showed highly significant positive correlations with both upper epidermis thickness and spongy tissue thickness (*p* < 0.01), while palisade tissue thickness was significantly positively correlated with stomatal width (*p* < 0.01). Physiologically, malondialdehyde (MDA) content demonstrated highly significant positive correlations with three antioxidant enzyme activities (*p* < 0.01), and catalase (CAT) activity showed highly significant positive correlations with peroxidase (POD) activity, soluble sugar content, and soluble protein content (*p* < 0.01). In *C. sasanqua*, tight coupling was observed between leaf structural parameters and stomatal characteristics: leaf thickness (Lth) showed highly significant positive correlations with both stomatal aperture (SA) and stomatal width (SW) (*p* < 0.01); upper epidermis thickness (UET) correlated significantly with SA, stomatal length (SL), and SW; while both lower epidermis thickness (LET) and spongy tissue thickness (ST) maintained highly significant positive correlations with SW (*p* < 0.01). For physiological aspects, MDA content showed highly significant correlations with CAT activity, superoxide dismutase (SOD) activity, and proline (Pro) content.

**Figure 10:**
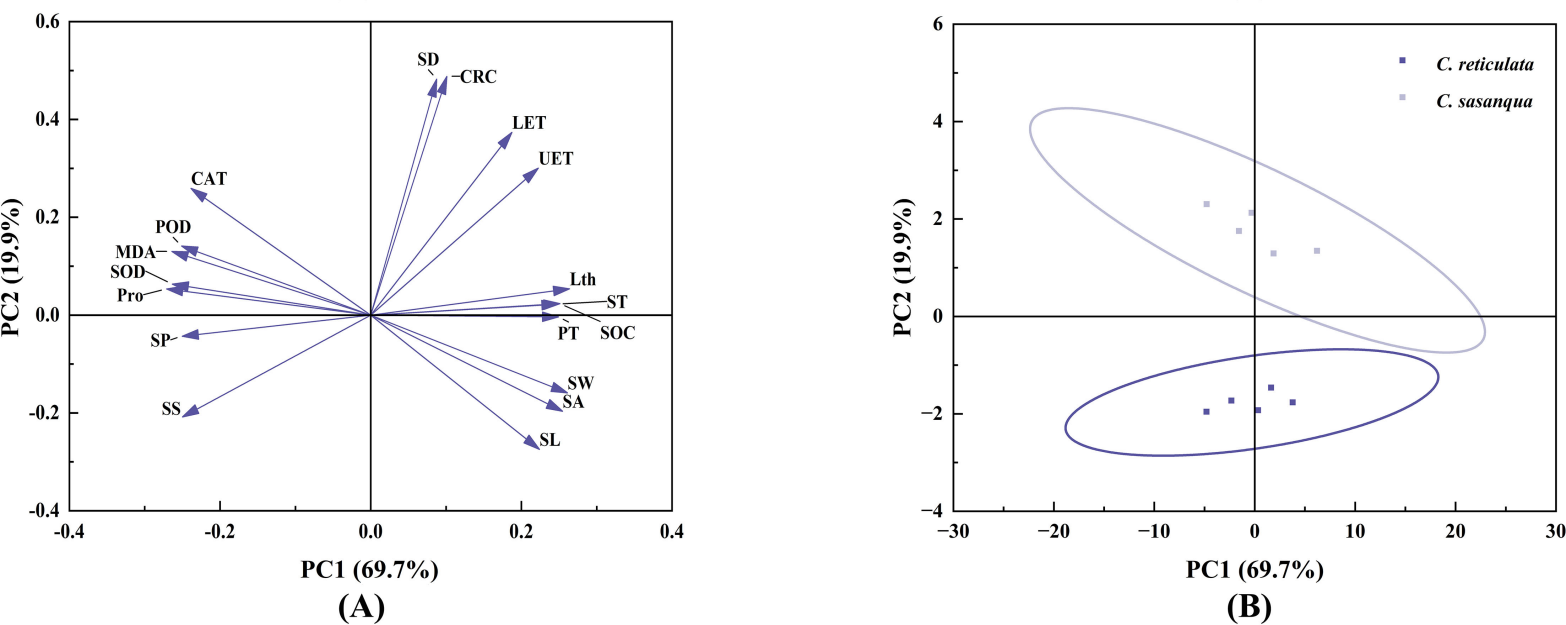
Principal component analysis (PCA) of 18 evaluation parameters for two *Camellia* species under drought stress. **(A)** Parameter distribution generated by PCA and their relative contributions to principal components (PC1) and (PC2). **(B)** PCA score plot showing the separation of the two *Camellia* species along principal components (PC1 and PC2).

To comprehensively and objectively evaluate the drought resistance of the two *Camellia* species, principal component analysis (PCA) was performed on 18 indicators. As shown in **Table 1**, the contribution rates of the first three principal components were 69.7%, 19.9%, and 3.7%, respectively, with a cumulative contribution rate reaching 93.3%. The cumulative variance contribution rate of the first three principal components exceeded 85%, indicating that the following indicators can serve as comprehensive metrics for evaluating drought resistance in both *Camellia* species. As shown in (**Figure 10**), the considerable separation in scatter plot distribution between the two *Camellia* species demonstrates substantial divergence in their drought resistance capabilities.

**Table 1:** Loading matrix of factors, eigenvalues, variance percentages, and contribution rate.

Indicators	Principal components		
	PC1	PC2	PC3
Lth	0.264	0.054	0.372
UET	0.222	0.301	0.069
LET	0.188	0.374	0.015
PT	0.249	-0.003	0.537
ST	0.249	0.022	0.251
SD	0.088	0.482	-0.171
SA	0.255	-0.197	0.078
SL	0.224	-0.274	-0.160
SW	0.261	-0.159	0.087
SOC	0.251	0.023	0.168
MDA	-0.264	0.130	0.233
CRC	0.101	0.488	-0.138
CAT	-0.239	0.259	0.153
POD	-0.251	0.141	0.323
SOD	-0.263	0.063	0.006
Pro	-0.271	0.053	0.066
SS	-0.250	-0.208	0.096
SP	-0.250	-0.043	0.441
Eigenvalue	12.550	3.576	0.662
Percentage of Variance (%)	69.720	19.868	3.675
Cumulative (%)	69.720	89.588	93.263

Lth, Leaf thickness; UET, Upper epidermis thickness; LET, Lower epidermis thickness; PT, Palisade tissue thickness; ST, Spongy tissue thickness; SD, Stomatal density; SA, Stomatal aperture; SL, Stomatal length; SW, Stomatal width; SOC, Stomatal opening count; MDA, Malondialdehyde; CRC, Chlorophyll relative content; CAT, Catalase; POD, Peroxidase; SOD, Superoxide dismutase; Pro, Proline; SS, Soluble sugar; SP, Soluble protein.

For the 18 measured indicators, principal component analysis was employed to extract common factors with a cumulative variance contribution rate ≥ 85%. Factor scores for different drought resistance indicators were then calculated. Using the variance contribution rates of the principal components as weights, the extracted scores were weighted and summed via membership function analysis to determine the coefficients of each indicator in the comprehensive drought resistance scoring model. The weights of the three principal components were calculated as 0.748, 0.213, and 0.039, respectively. The comprehensive evaluation value (D) and drought resistance ranking were subsequently derived. The comprehensive evaluation results are presented in **Table 2**, showing that the drought resistance followed the order: *C. reticulata*< *C. sasanqua*.

**Table 2:** The comprehensive indicator values, weights, membership function values, D values and comprehensive evaluation.

Species	Treatments	μ(1)	μ(2)	μ(3)	D	Average D value	Rank
*C. reticulata*	control	0.780	0.045	0.784	0.780	0.338	2
	T1	0.585	0.116	0.679	0.585		
	T2	0.466	0.007	0.715	0.466		
	T3	0.225	0.054	0.171	0.225		
	T4	0.000	0.000	0.416	0.000		
*C. sasanqua*	control	1.000	0.775	0.626	1.000	0.551	1
	T1	0.607	0.762	0.000	0.607		
	T2	0.408	0.958	0.340	0.408		
	T3	0.293	0.870	0.562	0.293		
	T4	0.000	1.000	1.000	0.000		

μ(x), subordinative function; D, comprehensive evaluation value.

## 4 Discussion

### 4.1 Effects of drought stress on the morphological characteristics of two *Camellia* species

Under drought stress, changes in leaf morphology serve as the most visually apparent manifestation. As drought intensity increases, the overall leaf area decreases, which is an adaptive mechanism for plants to reduce water transpiration from the leaf surface (Nardini, 2022). Leaf relative water content (RWC) reflects the degree of plant water deficit to a certain extent and provides a more intuitive indication of changes in plant water status. In this study, the RWC of the leaves of *C. reticulata* and *C. sasanqua* decreased with increasing drought stress, accompanied by varying degrees of wilting, curling, and discoloration. The leaves of *C. reticulata* showed severe drooping and loss of luster, while those of *C. sasanqua* demonstrated significant curling and turned withered yellow. Previous studies have indicated that drought stress affects plant deformation, and plants under severe water deficiency exhibit varying symptoms of drought damage, ranging from mild manifestations such as drooping branches and wilting or curling leaves to severe outcomes uncluding death (Li et al., 2022; Zahedi et al., 2025), which is largely consistent with the findings of this study.

### 4.2 Leaf anatomical structure

When plants are subjected to drought stress, their leaf epidermal structure, palisade and spongy tissue, leaf thickness, veins, and stomata undergo a series of adaptive responses (Yavas et al., 2024). Under drought conditions, the thickness of the spongy and palisade tissues in *Malus pumila* leaves decreases to varying degrees (Sun et al., 2022). In this study, as drought stress intensified, soil water content was positively correlated with leaf thickness, upper epidermal thickness, and the thinkness of palisade and spongy tissues in both camellias. Both species responded to drought stress by modulating leaf thickness. Leaf thickness is primarily determined by the upper and lower epidermis, palisade tissue, and spongy tissue. The observed reduction in leaf thickness may be attributed to decreased cellular water content under drought stress, which impairs normal growth and cell division, leading to diminished leaf growth, reduced cell size, and structural alerations, ultimately resulting in thinner leaves. Under well-watered conditions, the palisade tissue in rice *Oryza sativa* leaves maintains structural integrity and functional activity, significantly enhancing transpirational water loss. Conversely, during drought stress, this tissue reduces the rate of internal water evaporation to the environment, thereby effectively mitigating water loss. This adaptive response plays a crucial role in maintaining cellular water balance, improving plant drought tolerance, and sustaining physiological activity (Basu et al., 2016). In this study, when both camellias were subjected to drought stress at different stages, the thickness of their palisade and spongy tissues decreased significantly compared to the control, which was the main reason for the notable reduction in overall leaf thickness. Additionally, the mesophyll structure of both camellias became tighter, with decreased compactness and increased looseness. Under drought conditions, soil water content in *C. sinensis* showed a positive correlation with leaf thickness, upper and lower epidermal thickness, palisade and spongy tissue thickness, and relative leaf water content. Specifically, as soil water content looseness exhibited a negative correlation (Qian et al., 2018; Guo et al., 2017). These findings are largely consistent with previous research results.

Plants regulate photosynthesis and transpiration through the opening and closing of stomata. Under non-drought conditions, stomata remain open to facilitate gas exchange, enhancing photosynthesis and improving water-use efficiency. However, when subjected to drought stress, plants reduce water transpiration by adjusting stomatal aperture, density, and shape, thereby enhancing drought resistance. This represents a critical adaptive mechanism for coping arid environments (Xiang et al., 2021; Zhang et al., 2024; Zhao et al., 2015). The perception of drought signals and their initial transduction represent the primary steps in triggering stomatal closure (Soma et al., 2023). When root systems detect soil water deficit, they rapidly synthesize abscisic acid (ABA), which is subsequently transported to aerial tissues via the xylem, establishing ABA as a core signaling molecule mediating drought responses (Belfiore et al., 2021). As leaf ABA concentrations increase, ABA specifically binds to the PYR/PYL/RCAR receptor family on the guard cell plasma membrane. This binding inhibits type 2C protein phosphatases (PP2Cs, e.g., ABI1), thereby relieving their suppression of SnRK2 kinases and activating downstream signaling cascades that ultimately induce stomatal closure (Chen et al., 2021; Mukherjee et al., 2023). The activated SnRK2s precisely regulate ion fluxes (e.g., K^+^, Cl^-^, and Ca^2+^) across the guard cell plasma membrane through phosphorylation of ion channels. This orchestrated ion transport maintains cellular turgor pressure under drought stress, coordinately controls stomatal movement, minimizes water loss, and optimizes water use efficiency (Sato et al., 2009). In this study, both *Camellia* species exhibited a reduction in stomatal size and aperture with increasing drought severity; however, they displayed distinct patterns in stomatal density regulation, leading to divergent water conservation strategies. *C. reticulata* demonstrated an initial increase followed by a decrease in stomatal density, relying primarily on a significant reduction in stomatal aperture width to conserve water. In contrast, *C. sasanqua* showed a progressive decline in stomatal density throughout the stress period, achieving transpiration control mainly by reducing stomatal aperture length. These divergent strategies suggest that even closely related species within the same genus may employ distinct combinations of morpho-physiological adjustments to cope with environmental constraints. The initial increase in stomatal density in *C. reticulata* may represent a short-term acclimation mechanism to sustain gas exchange capacity during the early stages of water limitation. Conversely, the consistent down-regulation of stomatal density in *C. sasanqua* signifies a more conservative and stable water-saving strategy, which likely constitutes a key physiological basis for its reportedly greater adaptability in arid habitats. Previous studies have demonstrated that *C. reticulata* response resembles that of *Ziziphus jujuba* (Shipeng, 2006) while *C. sasanqua* pattern aligns with *C. oleifera* ‘Wu 2’ (He et al., 2020). collectively underscore the complexity of stomatal regulatory networks. These networks do not adhere to a singular model but exhibit remarkable species specificity. Similar to how *Poa pratensis L*. enhances drought resistance by reducing stomatal density (Liu et al., 2009). the coordinated down-regulation strategy observed in *C. sasanqua* in this study may represent a more optimized adaptive solution under persistent drought pressure.

Previous research on *C. sinensis* has demonstrated that drought stress induces the formation of sparse wax crystals on the leaf surface, which compromises the cuticle’s ability to restrict water movement, confer drought resistance, and prevent organ fusion (Chen et al., 2020). In this study, comparative analysis of leaf surface characteristics in two ornamental *Camellia* species revealed a general enhancement of wax accumulation under progressive drought stress. This observation aligns with established findings that plants often increase epicuticular wax deposition as an adaptive mechanism to improve drought tolerance. Substantial evidence indicates that the synthesis, transport, and structural integrity of plant epicuticular wax are governed by complex genetic networks. In *Arabidopsis thaliana*, AP2/DREB family transcription factors DREB26 and RAP2.4 directly bind to the promoters of key wax biosynthesis genes *KCS2* and *CER1*, activating their expression and promoting wax deposition (Yang et al., 2020). The drought-induced MYB96 transcription factor further enhances wax accumulation and drought tolerance by regulating *KCS1*, *KCS2*, and *KCS6* (Seo and Park, 2011). In rice (*Oryza sativa*), the ethylene-responsive factor OsWR1 recognizes and binds to DRE/GCC cis-elements in the promoters of *OsLACS2* and *OsFAE1*, positively regulating wax biosynthesis. Overexpression of *OsWR1* significantly improved drought tolerance at the seedling stage, whereas RNAi lines exhibited reduced wax content and compromised stress resistance (Wang et al., 2012; Zhu and Xiong, 2013). Similarly, the drought-induced gene *DWA1* (drought-induced wax accumulation 1) positively regulates cuticular wax accumulation, with mutants showing decreased wax content and diminished drought tolerance. In maize (*Zea mays*), *GL6* (glossy6) facilitates intracellular wax transport, and its loss-of-function mutant displays reduced wax deposition, increased cuticular permeability, accelerated water loss, and significantly impaired seedling drought tolerance (Li et al., 2019). *ZmSRL5* (semi-rolled leaf 5) maintains cuticular barrier function by modulating wax structure and spatial distribution; mutants exhibit uneven wax distribution, structural defects, and exacerbated water loss, leading to reduced drought resistance (Pan et al., 2020). Drought stress commonly upregulates epicuticular wax coverage in plants. The composition, abundance, structure, and effective deposition of wax collectively determine the barrier efficacy of the cuticle, thereby influencing plant water retention capacity and drought performance. These studies consistently highlight epicuticular wax not only as a crucial component of the physical barrier but also as a central regulatory node in plant responses to abiotic stresses, particularly drought (Kim et al., 2019; Oliveira et al., 2003). During the early drought stage, the leaf surfaces of *C. reticulata* exhibited increased roughness with wax accumulating in clustered and honeycomb-like structures. This was followed by a marked reduction in wax coverage during the mid-stress period, before demonstrating significant re-accumulation in the late stress phase. In contrast, *C. sasanqua* displayed minimal wax deposition with striated distribution patterns initially, which progressively intensified from the mid-stress stage onward, ultimately peaking with dense, striated wax formations during late stress. Throughout the stress regime, *C. sasanqua* maintained significantly higher epicuticular wax coverage than *C. reticulata*, suggesting enhanced cuticular barrier function and potential drought resistance advantages.

### 4.3 Leaf physiology and biochemistry

Drought stress inhibits the increase in relative chlorophyll content in both *Camellia* species. Photosynthesis serves as the fundamental basis for plant growth, with chlorophyll acting as the primary photosynthetic pigment driving this process. Drought stress reduces chloroplast pigment content, manifesting as yellowing or reddening of leaves. This adjustment helps alleviate photosynthetic pressure and protect the leaves from damage caused by drought stress (Çakmakçı and Güner, 2024). Zhao et al. (2019) investigated the effects of drought stress on the growth and chlorophyll fluorescence characteristics of *Lonicera japonica*, finding that increase drought severity significantly decreased chlorophyll content, which is largely consistent with the conclusions of this study.

*Camellia* species deploy sophisticated physiological mechanisms to cope with drought stress, including osmotic regulation, reactive oxygen species (ROS) scavenging, and phytohormonal signaling (Das et al., 2015; Liu et al., 2015). Among these responses, the dynamic accumulation of malondialdehyde (MDA) serves as a critical indicator of membrane system integrity, reflecting the progression of oxidative damage under drought conditions while demonstrating close correlation with stomatal behavior and ROS metabolism (Khamis et al., 2025). Drought stress activates the abscisic acid (ABA) signaling pathway, subsequently triggering substantial ROS production (Cassells and Curry 2001). The excessively accumulated ROS initiates membrane lipid peroxidation, leading to significant MDA accumulation. As a highly reactive aldehyde, MDA can further exacerbate cellular damage by cross-linking with biological macromolecules including proteins and nucleic acids, thereby disrupting membrane protein structure and enzymatic activities (Zheng et al., 2024). Moreover, excessive ROS accumulation can induce lipid peroxidation, protein degradation, enzyme inactivation, and broadly compromise nucleic acid stability alongside various intracellular components, ultimately potentially leading to programmed cell death (Upadhyaya et al., 2008).

MDA is one of the products of membrane lipid peroxidation in plant cells. Drought stress causes water loss in plant cells, disrupts biomembrane structures and leads to the production of MDA. As a byproduct of membrane lipid peroxidation or delipidation, its accumulation can cause oxidative damage to cells and impair normal plant growth and development. Therefore, the MDA content serves as a key indicator of the extent of cellular damage—higher levels indicate more severe damage (Cui et al., 2018). Studies have found that under drought stress, the MDA content in *C. sinensis* shows a continuous increasing trend (Qian et al., 2018). This aligns with the conclusions of the present study, as intensified drought conditions inhibit cell division and expansion, slow down plant metabolism and osmotic regulation, and exacerbate membrane lipid peroxidation, leading to electrolyte leakage and consequently, significant MDA accumulation (Barros et al., 2021).

Under drought stress, plants respond to adversity by accumulating osmoregulatory substances substances such as soluble sugars, proline, and soluble proteins. These compounds help reduce cellular fluid concentration, prevent cell dehydration, and thereby enhance the plant’s drought resistance (Imran et al., 2024). This study found that as drought stress intensified, the soluble protein content, proline content, and soluble sugar concentration increased in both *Camellia* species. Related research (Shen et al., 2022) on *C. vietnamensis* has shown that soluble sugar concentration, proline content, and soluble protein content significantly rise with increasing drought severity. Furthermore, the increase in proline content across all drought stages was more pronounced in both camellias compared to soluble sugars and soluble proteins. Therefore, it can be preliminarily inferred that proline is a key osmotic substance determining the drought tolerance of camellias.

Under drought stress, the primary mechanism of plant antioxidant defense involves SOD, CAT, and POD, which effectively mitigate damage to cells caused by reactive oxygen species (Bhardwaj and Yadav, 2012). Within the antioxidant metabolic system, SOD, CAT, and POD serve as key protective enzymes in plants facing abiotic stress. They play crucial roles in scavenging biological free radicals, and their activity is closely linked to the plant’s adaptability to adverse conditions (Liying et al., 2005). Therefore, the activities of SOD, CAT, and POD in plants are coordinated to respond to drought stress. In this study, both *C. reticulata* and *C. sasanqua* exhibited significantly higher activities of SOD, CAT, and POD under drought stress compared to the control group. Moreover, as drought severity increased, the activities of these enzymes showed a proportional rise. Related research (Guo et al., 2023) on *C. oleifera*, also demonstrated that SOD, CAT, and POD activities were significantly higher under increasing drought conditions campared to the control, However, some studies have reported conflicting results. For instance, one investigation (Lin et al., 2023) using PEG-6000 simulated drought stress on *C. reticulata* found that POD activity initially increased but subsequently decreased with progressing drought severity. This discrepancy may be attributed to the drought intensity exceeding the plant’s defensive capacity, leading to reduce antioxidant enzyme activity, enhanced cellular peroxidation, and increased generation of free radicals (Mishra et al., 2023).

### 4.4 Comprehensive evaluation

This study analyzed the correlation among various indicators of the two *Camellia* species and preliminarily explored their differences in drought resistance. Principal component analysis (PCA) was employed to extract key information through linear dimensionality reduction, while the membership function method was used to process fuzzy data and support multi-category membership (Wang et al., 2025). Given the complexity of abiotic stress evaluation in plants and its species-specific nature, this study utilized PCA to assess the drought tolerance of the two camellias. The order of drought tolerance was determined as: *C. reticulata*< *C. sasanqua*.

## 5 Conclusions

As the severity of drought stress intensified, the leaf morphological characteristics of both *Camellia* species exhibited varying degrees of change. The results demonstrated that the leaf relative water content, leaf thickness, lower epidermis thickness, palisade tissue thickness, stomatal density, stomatal aperture, stomatal length, stomatal width, and relative chlorophyll content all showed a gradual declining trend. In contrast, leaf surface wax content, MDA, proline, soluble sugars, soluble proteins, as well as the activities of SOD, CAT, and POD displayed an increasing trend. This study employed principal component analysis to comprehensively evaluate the drought tolerance of the two *Camellia* species, with results indicating the following order of drought resistance: *C. reticulata*< *C. sasanqua*. Future research should focus on the molecular mechanisms underlying these observed physiological differences, particularly the transcriptional regulation of wax biosynthesis *CER1, KCS* genes and stomatal development. Further investigation is warranted to explore the long-term performance of these species under field conditions, integrating variable factors like recovery capacity and interactions with other abiotic stresses.

## Data Availability

The original contributions presented in the study are included in the article/supplementary material, further inquiries can be directed to the corresponding author/s.
